# Prevalence characteristics of human papillomavirus (HPV) infection among women receiving physical examinations in the Shangcheng District, Hangzhou city, China

**DOI:** 10.1038/s41598-021-96131-y

**Published:** 2021-08-16

**Authors:** Lu Wang, Chunfeng Yu, Xiaoling Ni, Fang Wang, Caihe Wen, Mei Jin, Juanjuan Chen, Kunming Zhang, Jiahao Wang

**Affiliations:** 1 Department of Obstetrics and Gynecology, Hangzhou Women’s Hospital, Hangzhou, China; 2 Department of Clinical Laboratory, Affiliated Hangzhou Cancer Hospital, Zhejiang University School of Medicine, Hangzhou, China; 3 Department of Radiation Oncology, Affiliated Hangzhou Cancer Hospital, Zhejiang University School of Medicine, Hangzhou, China

**Keywords:** Public health, Cancer screening

## Abstract

This paper aimed to investigate the characteristics of female HPV infection in the Shangcheng District, Hangzhou city, China. The retrospective study was designed to analyze the HPV prevalence rate of 22,382 women receiving physical examinations from 2016 to 2020 in the Shangcheng District of Hangzhou city in China. A commercial kit was designed to detect the HPV genotypes. Trends were examined for age-specific groups (≤ 30 years, 31–44 years, 45–54 years, 55–64 years,  ≥ 65 years). A receiver operating characteristic (ROC) analysis was used to assess the correlation of age classification in high risk HPV (HR-HPV) infection. 22.41% (5015/22,382) of samples were HPV positive, 91.28% (4578/5015) of HPV positive women were infected by HR-HPV. The most prevalent HR-HPV genotypes were 16, 52, 18, 58, 56, and 51. The trend of HPV prevalence showed the significant differences in age-specific groups (χ^2^ = 164.70, P < 0.001). Moreover, the areas under ROC curve (AUC) was 0.712 in 55–64 years group which showed a strong contribution of age classification for HR-HPV infection. This study provided baseline data on the prevalence characteristics of HPV infection and the critical age group of HR-HPV prevalence rate was 55–64 y among the samples receiving physical examinations.

## Introduction

Cervical cancer is one of the major malignant tumors threatening women all over the world. Persistent infection of high-risk human papillomavirus (HR-HPV) is a necessary factor for the occurrence of cervical cancer^1,2^, however, it’s prevalence and genotype distribution were various in different countries worldwide^3–8^. More than 100 HPV genotypes have been identified, which are classified into high risk (HR) and low risk (LR) types on the basis of their correlation with cervical cancer^9–11^. HPV (16, 18, 31,33, 35, 39, 45, 51, 52, 53, 56, 58, 59, 66, 68, 73) and HPV (6, 11, 40, 42, 43, 44,54, 61, 70, 72, 81,82,83) were clearly classified by International Agency for Research on Cancer (IARC) as common high risk types and low risk types^12,13^.

It is difficult to establish an accurate database of the prevalence and genotype distribution for HR-HPV infection in China because of the immense geographical area and the largest population. There is still lack of comprehensive data among women receiving physical examinations on the prevalence and genotype distribution of HPV infection in Hangzhou. The objective of this study was to investigate the characteristics of the distribution of HPV genotypes among women with different age group living in Hangzhou and provide a theoretical basis for cervical cancer screening in this region.

## Results

### Overall prevalence of HPV infections

Of 22,382 subjects, 5015 were positive for HPV infection, with the infection rate of 22.41%. The HPV prevalence rates in five age groups were 20.84%, 18.83%, 21.28%, 28.08% and 20.98%, respectively. Chi-squared test showed the significant differences in HPV infection among age-specific groups and the highest HPV infection rate was found in age group of 55–64 y with the prevalence rate of 28.08% (χ^2^ = 164.70, P < 0.001) (Table 1).

**Table 1:** Positive rate of women receiving physical examinations in age-specific groups.

Age (y)	No Positive cases	No Negative cases	No Total	Positive rate (%)
≤ 30	476	1808	2284	20.84
31–44	1095	4720	5815	18.83
45–54	1301	4811	6112	21.28
55–64	1695	4341	6036	28.08
≥ 65	448	1687	2135	20.98
Total	5015	17,367	22,382	22.41

### Age-specific HR-HPV prevalence

In the overall HPV infection population, 91.28% of women had HR-HPV infection. The high risk proportions in 5 age groups were 88.45%, 91.51%, 91.78%, 91.74% and 90.63%, respectively. Chi-squared test indicated no significant differences in HR-HPV infection among age-specific groups (χ^2^ = 5.97, P = 0.201) (Table 2).

**Table 2:** High risk proportion of each age group.

Age (y)	No High risk infection	No Low risk infection	No Total	High risk proportion (%)
≤ 30	421	55	476	88.45
31–44	1002	93	1095	91.51
45–54	1194	107	1301	91.78
55–64	1555	140	1695	91.74
≥ 65	406	42	448	90.63
Total	4578	437	5015	91.28

### HPV genotype distribution

**Figure 1:**
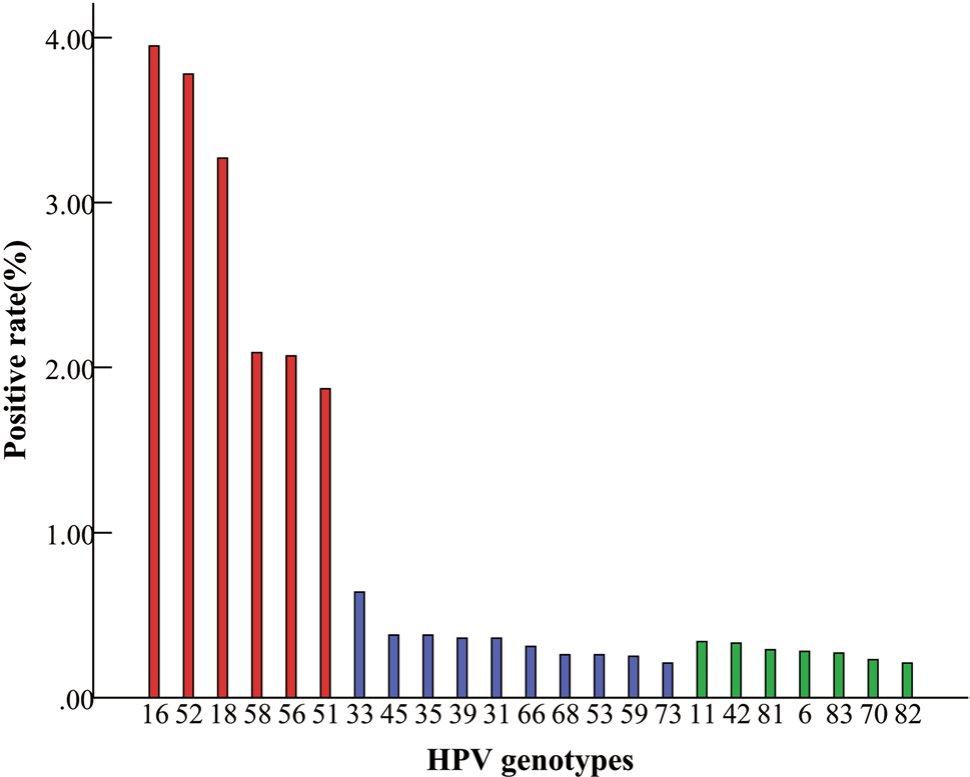
The prevalence and genotype distribution of HPV among 22,382 women. (Red columns represented the most prevalent HR-HPV genotypes, blue columns represented the other HR-HPV genotypes and green columns represented the LR-HPV genotypes). This figure was created by the SPSS software, version 19.0.

Figure 1 showed the distribution of HPV genotypes in all the enrolled women. The prevalence of HR-HPV and LR-HPV infections was 20.45% (4578/22,382) and 2.00% (437/22,382), respectively. The most commonly detected HR-HPV types were HPV-16, HPV-52, HPV-18, HPV-58, HPV-56, and HPV-51 with the overall proportions of 3.95%, 3.78%, 3.27%, 2.09%, 2.07% and 1.87% in samples, respectively.

In age-specific groups, HPV-52 was highest detected in the ≤ 30 y (4.51%), 45–54 y (3.29%) and ≥ 65 y (3.95%) age groups, HPV-16 was commonly detected in the 31–44 y (3.35%) and 55–64 y (5.86%) age groups (Table3). The prevalence of HPV-16 (5.86%) and HPV-52 (4.54%) in the 55–64 y age groups was statistically higher than the other age-specific groups (χ^2^ = 90.17, P < 0.001).

**Table 3:** HPV prevalence and genotype distribution.

	≤ 30 y (n = 2284)		31–44 y (n = 5815)		45–54 y (n = 6112)		55–64y (n = 6036)		≥ 65y (n = 2135)	
HR-HPV types	Positive cases	Prevalence (%)	Positive cases	Prevalence (%)	Positive cases	Prevalence (%)	Positive cases	Prevalence (%)	Positive cases	Prevalence (%)
52	103	4.51	184	3.16	201	3.29	274	4.54	84	3.95
16	76	3.33	195	3.35	185	3.03	354	5.86	73	3.42
58	31	1.36	116	1.99	126	2.06	152	2.52	42	1.97
56	35	1.53	129	2.22	115	1.88	145	2.40	41	1.92
18	84	3.68	166	2.85	184	3.01	227	3.76	72	3.37
51	20	2.23	84	1.44	102	1.67	182	3.02	31	1.45
66	8	0.35	20	0.34	22	0.36	14	0.23	5	0.23
33	10	0.44	25	0.43	58	0.95	47	0.78	4	0.19
31	5	0.22	11	0.19	33	0.54	22	0.36	9	0.42
59	8	0.35	8	0.14	22	0.36	16	0.27	3	0.14
45	9	0.39	15	0.26	35	0.57	17	0.28	8	0.37
35	14	0.61	14	0.24	39	0.64	14	0.23	5	0.23
39	5	0.22	10	0.17	20	0.33	36	0.60	9	0.42
68	4	0.18	6	0.10	23	0.38	18	0.30	7	0.33
53	4	0.18	6	0.10	25	0.41	21	0.35	3	0.14
73	5	0.22	13	0.22	4	0.07	16	0.27	10	0.47
**LR-HPV types**										
6	12	0.53	13	0.22	9	0.15	21	0.35	7	0.33
11	10	0.44	21	0.36	25	0.41	16	0.27	5	0.23
42	6	0.26	17	0.29	15	0.25	26	0.43	10	0.47
70	0	0.00	6	0.10	26	0.43	13	0.22	6	0.28
81	14	0.61	19	0.33	3	0.05	22	0.36	7	0.33
82	0	0.00	9	0.15	17	0.28	19	0.31	3	0.14
83	13	0.57	8	0.14	12	0.20	23	0.38	4	0.19

### ROC curve analysis for age classification of HR-HPV infection

**Figure 2:**
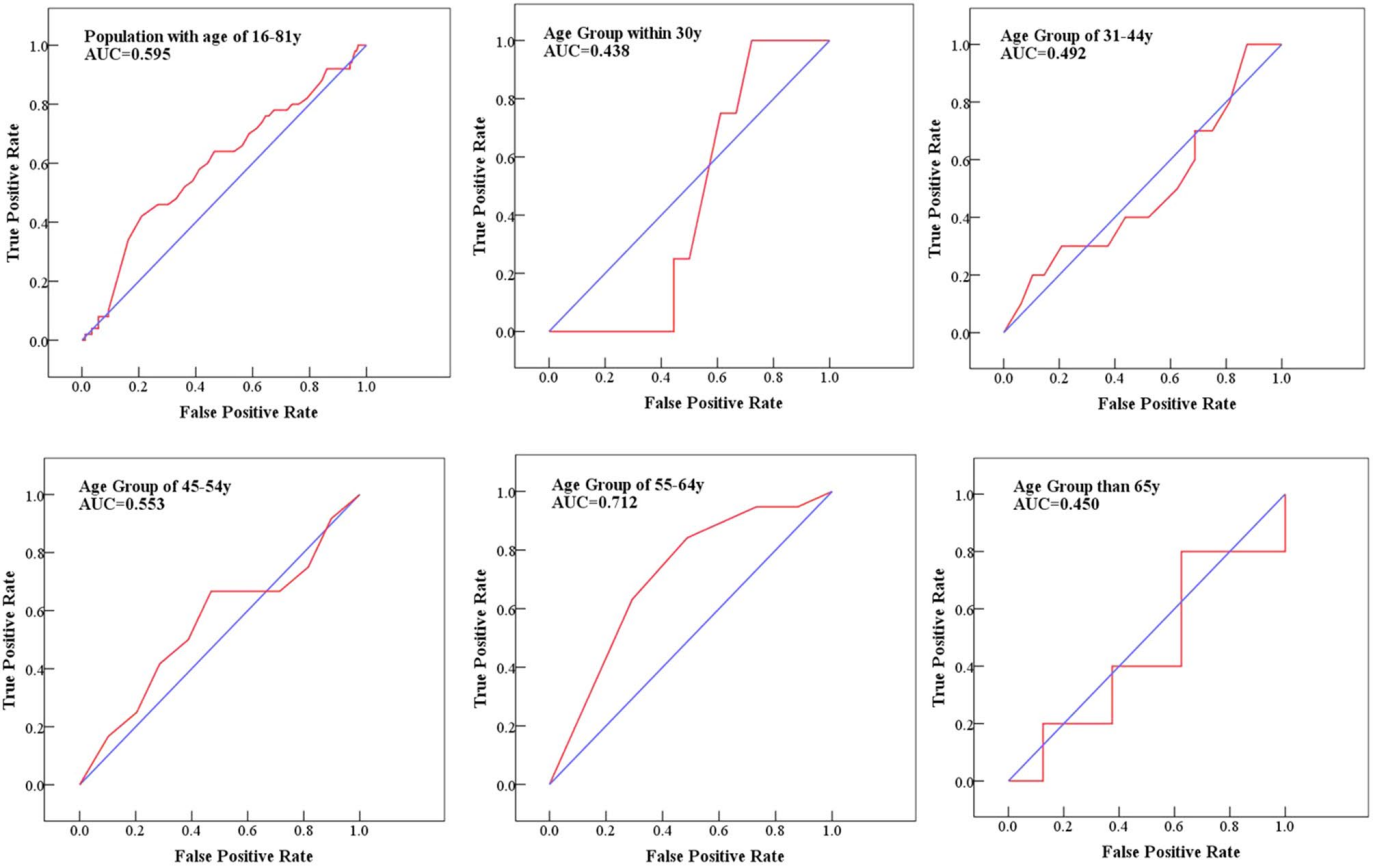
ROC curve and AUC values for HR-HPV infection with age-specific groups (The blue curve represented the random chance and red curve represented the sensitivity curve which means the more closer to the top left, the more accurate to the test). This figure was created by the SPSS software, version 19.0.

Table 4 showed the metrics of ROC curve for the samples and age-specific groups. The population with age of 16–81 y achieved an AUC of 0.595 (P = 0.041) for HR-HPV infection, and 0.438, 0.492, 0.553, 0.712 and 0.450 for age groups of ≤ 30 y (P = 0.702), 31–44 y (P = 0.934), 45–54 y (P = 0.574), 55–64 y (P = 0.009) and ≥ 65 y (P = 0.741), respectively. The comparison of ROC curves between age-specific groups were shown in Fig. 2. The age group of 55–64 y had a strong contribution to HR-HPV infection.

**Table 4:** The metrics of ROC curve for the samples and age classification.

Age (y)	AUC	95% CI	P
≤ 30	0.438	0.210–0.665	0.702
31–44	0.492	0.288–0.695	0.934
45–54	0.553	0.358–0.747	0.574
55–64	0.712	0.574–0.849	0.009
≥ 65	0.450	0.150–0.750	0.741
16–81	0.595	0.503–0.688	0.041

## Discussion

HPV is associated with varied epithelial lesions, including cervical intraepithelial neoplasia and genital warts^15^. HPV detection has been proposed by European Guidelines in several applications including primary screening, triage test of equivocal cytological results and follow up with women treated for CIN^16,17^. Knowledge of HPV prevalence and genotype is also important for characterization of the population in HPV vaccination trials and monitoring the efficacy of the vaccine^18^. Compared with region-based data on the Chinese population of HR-HPV positive rates, a meta-analysis that summarised the reported data for Haikou (31.9%), Guangdong (20.0%), Chongqing (27.3%), Jinan (25.7%), Shanghai (22.6%) and Nanchang (18.4%)^19^. The present study showed HPV positive rate among women in the Shangcheng District of Hangzhou city in China was 22.41% and the HR-HPV positive rate was 20.45% which might be lower or similar compared with the above report. However, different HPV prevalence rates may be a combination of different cultural diversity, survey periods, geographic locations, and economic levels. The cervical cancer screening practices are heterogeneous and inadequate implementation even in Europe^33^, and the population-based organized cervical cancer screening has been completed in only 9 of 28 European countries^34^ which maybe more difficult in China because of geographic and population diversity.

HPV-16, HPV-18, HPV-31, HPV-58 and HPV-52 were the five most common HR-HPV types worldwide, HPV-16 and HPV-18 account for approximately 70% of cervical cancer cases^20^. In China, HPV-16, HPV-58 and HPV-52 were the top three most common types in most regions^21,22^. Analysis of HPV prevalence showed that the positive rate of HPV-16 was still the highest in our research, accounting for 3.95% of the total population (884/22,382). The top six HR-HPV types were 16,52,18,58,56and 51, which were the same as those reported in several domestic studies^23–25^. Figure 1 and Table3 showed the distribution of HPV genotypes in all the samples, HPV-52 and HPV-16 were commonly detected in age-special women groups (7.7%, 1729/22,382). Furthermore, HPV-52 (3.78%) might have a slight higher prevalence rate in our study than HPV-18 (3.27%), which was more common in other countries in the world^14^. These findings indicated that in addition to HPV-16 and HPV-18, an HPV vaccine in Hangzhou should include HPV-51, 52, 56 and 58 genotypes.

The HPV infection rates in different age groups were also varied. Previous studies have showed that HPV prevalence decreased with the increasing age worldwide^26,27^, however, two peaks of age-specific prevalence of HPV including younger and older women were also revealed in other reports^28–30^. Various conclusions of HPV infection were usually generated by heterogeneous study cohorts including inpatients or outpatients, indeed, women receiving physical examinations also play an important role in epidemiological investigation of HPV infection. In our study, chi-squared test showed the significant differences in HPV infection among age-specific groups and the highest HPV infection rate was found in age group of 55–64 y with the prevalence rate of 28.08% (χ^2^ = 164.70, P < 0.001), and the test of HR-HPV infection was no significant differences among age-specific groups (χ^2^ = 5.97, P = 0.201). Although the peak age of HPV infection in different studies was diverse, the peak occurred primarily in perimenopause women which may be due to physiologic and immunologic dysregulation^31^. Of all population receiving physical examinations, the prevalence of HPV-16 and 52 in the 55–64 y age groups (5.86% and 4.54%) was statistically higher than the others (χ^2^ = 90.17, P < 0.001). The findings suggested that cervical cancer screening programs should pay more attention to the women cohorts of 55–64 y.

Age is an important factor associated with HR-HPV infection. The true positive rate and false positive rate of HR-HPV infection in age-specific groups were generated by ROC curve, and values of AUC were used to estimate the correlation between HR-HPV and ages. As showed in Fig. 2, the superior value of AUC which signified age group of 55–64 y had a strong contribution to HR-HPV infection in women receiving physical examinations.

The limitation of current study is absent analysis of mix HPV infection. The reason is that whether HPV mix infections increase the risk of disease is not clear and antigens vary among different HPV types, cross-defense is not effective enough^32^. Lack of individualized organized cervical cancer screening program for women receiving physical examinations including opportunistic cervical lesions is another limitation which will bring about the biased prevalence. Nevertheless, statistical methods were used to overcome these shortcomings.

In conclusion, we conducted a study with population receiving physical examinations on the prevalence of HPV and age-related groups in the Shangcheng District of Hangzhou city. The HPV prevalence and distribution varied significantly in different female age groups especially in 55–64 y. Prevention and screening strategies should be adjusted according to the HPV characteristics of age-special groups in this region.

## Materials and methods

### Study population

The retrospective study was designed to analyze the results of cervical HPV test of 22,382 women who receiving physical examinations in the Shangcheng District of Hangzhou city from October 2016 to November 2020. The mean age of the population was 43 years ranging from 16 to 81 years and the data was divided into 5 age groups (≤ 30 years, 31–44 years, 45–54 years, 55–64 years,  ≥ 65 years). The inclusion criteria for the study were as follows: women who were attending first-time cervical screening, living in the Shangcheng area, were not currently pregnant and had no history of total uterus or cervix resection. The exclusion criteria were the following: women who were in their second round of screening or more, receiving cervical physical therapy or had a history of any cancers. This study was approved by the Ethical Committee of Hangzhou Women’s Hospital. Informed consent was obtained from all participants including parents of 17 young people under the age of 18 years, and all methods were performed in accordance with the relevant guidelines and regulation.

### HPV genotyping

HPV detection and typing were performed on the collected specimens using the real-time polymerase chain reaction (RT-PCR) melt curve test by a commercial kit (Human Papillomavirus Genotyping Kit, Dian, Hangzhou, China) which can identify 16 HR-HPV types (16,18,31,33,35,39,45,51,52,53,56,58,59,66,68 and 73) and 7 LR-HPV types (6,11,42,70,81,82 and 83). The RT-PCR platform possesses the advantages of convenience, high throughput, low time and cost, and low risk of false positive results due to cross-contamination of PCR. The experimental conditions for RT-PCR Test followed the guidelines of associated protocols. Briefly, 23 HPV specific fragments were amplified by this kit, and the products were hybridized with the designed molecular beacon probe to collect fluorescence signals which could determine the HPV types according to the melting temperature (Tm) values.

### Population evaluation of HR-HPV infection based on the age classification

The age classification was chosen for the estimated metric of HR-HPV infection in population receiving physical examinations. ROC curve analysis was used to assess the performance between age classification and HR-HPV infection. AUC with a value of 1 indicates an ideal result, whereas values lower than 0.5 means an insignificant result.

### Statistical analysis

All statistical analyses were performed using SPSS V.19.0 (SPSS, Chicago, Illinois, USA). Chi-squared tests were used to assess the statistical significance of any differences in HR-HPV prevalence. P < 0.05 was considered statistically significant.
